# Genetic Ablation of the ClC-2 Cl- Channel Disrupts Mouse Gastric Parietal Cell Acid Secretion

**DOI:** 10.1371/journal.pone.0138174

**Published:** 2015-09-17

**Authors:** Meghali P. Nighot, Prashant K. Nighot, Thomas Y. Ma, Danuta H. Malinowska, Gary E. Shull, John Cuppoletti, Anthony T. Blikslager

**Affiliations:** 1 North Carolina State University, College of Veterinary Medicine, Raleigh, North Carolina, United States of America; 2 University of Cincinnati College of Medicine, Cincinnati, Ohio, United States of America; 3 University of New Mexico School of Medicine, Albuquerque, New Mexico, United States of America; Cinvestav-IPN, MEXICO

## Abstract

The present studies were designed to examine the effects of ClC-2 ablation on cellular morphology, parietal cell abundance, H/K ATPase expression, parietal cell ultrastructure and acid secretion using WT and ClC-2^-/-^ mouse stomachs. Cellular histology, morphology and proteins were examined using imaging techniques, electron microscopy and western blot. The effect of histamine on the pH of gastric contents was measured. Acid secretion was also measured using methods and secretagogues previously established to give maximal acid secretion and morphological change. Compared to WT, ClC-2^-/-^ gastric mucosal histological organization appeared disrupted, including dilation of gastric glands, shortening of the gastric gland region and disorganization of all cell layers. Parietal cell numbers and H/K ATPase expression were significantly reduced by 34% (*P*<0.05) and 53% (*P*<0.001) respectively and cytoplasmic tubulovesicles appeared markedly reduced on electron microscopic evaluation without evidence of canalicular expansion. In WT parietal cells, ClC-2 was apparent in a similar cellular location as the H/K ATPase by immunofluorescence and appeared associated with tubulovesicles by immunogold electron microscopy. Histamine-stimulated [H^+^] of the gastric contents was significantly (*P*<0.025) lower by *9*.4 fold (89%) in the ClC-2^-/-^ mouse compared to WT. Histamine/carbachol stimulated gastric acid secretion was significantly reduced (range 84–95%, *P*<0.005) in ClC-2^-/-^ compared to WT, while pepsinogen secretion was unaffected. Genetic ablation of ClC-2 resulted in reduced gastric gland region, reduced parietal cell number, reduced H/K ATPase, reduced tubulovesicles and reduced stimulated acid secretion.

## Introduction

ClC-2 is a broadly expressed Cl^−^ channel activated by hyperpolarization, extracellular (luminal) acidic pH, fatty acids including lubiprostone, amidation, acid-activated omeprazole and in some species (rabbit and human, but not mouse) protein kinase A [1–10]. It has also been suggested that ClC-2 can be activated by protein kinase C [11], consistent with the presence of many potential PKC phosphorylation sites in ClC-2 [12]. Other fatty acids have also been shown to activate acid secretion [13]. The ClC-2 Cl^-^ channel has been suggested to contribute to epithelial Cl^−^ secretion in human and rat airway epithelium, rat fetal lung, rat, mouse and pig intestinal epithelium, T84 cells and in rabbit gastric acid secretion [3,14–19]. Gastric mucosal parietal cells are highly differentiated and responsible for gastric acid production through the coordinated action of the H/K ATPase and apical Cl^-^ and K^+^ channel(s)/transporters [1,3,20–25]. Secretagogue stimulation results in a rapid, major morphological transformation in parietal cells, essential for maximal acid secretion to occur wherein cytoplasmic tubulovesicles containing H/K ATPase (and perhaps Cl^-^ and K^+^ channels/transporters) fuse with the apical membrane to form a greatly expanded secretory canaliculus with increased elongated microvilli which are then are recycled back during the resting stage [20,26,27]. Schofield, Ito and Bolender [27] showed that maximal acid secretion induced with histamine and carbachol occurs in tandem with maximal morphological rearrangement and that the changes in the tubulovesicles and apical membrane microvilli correlated with maximal acid secretion. Experimental conditions needed to obtain maximal parietal cell acid secretion and morphological transformation in the mouse stomach described in [27], were followed in the present studies.

Besides ClC-2 [3], several other Cl^-^ channels and transporters have been suggested to contribute to gastric Cl^-^ secretion, including the chloride intracellular channel-6 (CLIC-6) [28], cystic fibrosis transmembrane regulator (CFTR) [29], anion exchanger Slc26a9 [30], K^+^-Cl^-^ cotransporter-4 (KCC4) [31] and most recently ClC-5 [32]. ClC-2 was localized to the gastric parietal cell in isolated rabbit gastric glands where its location was similar to that of the H/K ATPase [33]. ClC-2 was associated with the parietal cell canalicular membrane and tubulovesicles by immunogold electron microscopy [33]. In contrast, other groups have suggested that ClC-2 is not associated with gastric HCl secretion since there was no difference in the pH of the gastric contents of WT and ClC-2^-/-^ mice after 15 min of histamine stimulation [34].

It is known that ClC-2 ablation in mice leads to disorganization and degeneration of retinal photoreceptors and male germ cells [34]. In intestine, ClC-2 plays an important role in regulating intestinal barrier function [17,35] and intestinal villus and apical tight junction structure were altered in the absence of ClC-2 [36,37]. Recently, ClC-2 was also demonstrated in porcine gastric mucosa and the ClC-2 agonist SPI-8811 (cobiprostone) rescued gastric mucosal barrier function and ameliorated acid-induced gastric injury [38]. Therefore the present studies were designed to investigate whether ClC-2 ablation also results in adverse effects on the gastric mucosa with a focus on parietal cell abundance, H/K ATPase expression, morphology and acid secretion using WT and ClC-2^-/-^ mouse gastric mucosa.

## Materials and Methods

Studies were all approved by the North Carolina State University and University of Cincinnati Institutional Animal Care and Use Committees. Mice were euthanized by CO_2_ asphyxiation using approved AVMA methods

### Materials

Histamine HCl, carbachol, thiobutabarbital (Inactin), Pefabloc, bestatin, aprotinin, leupeptin, pepstatin A and rabbit IgG gold secondary antibody were purchased from Sigma-Aldrich Inc (St Louis, MO). Diphenhydramine was from ICN Biomedicals (Irvine, CA). Mouse monoclonal H/K ATPase β1 subunit and rabbit β-actin antibodies were from Abcam (Cambridge, MA). Rabbit ClC-2 antibody was from Alomone Labs (Jerusalem, Israel). DAPI mounting medium and secondary antibodies conjugated with Alexa Fluor 488 or Cy3 were from InVitrogen (Eugene, OR). Biotinylated secondary antibody, avidin-substrate and peroxidase developing solutions were obtained from Vector Laboratories (Burlingame, CA). BCA Protein Assay Kit and luminol enhancer solution were from Pierce (Rockford IL). PVDF membranes were from Immobilon, Millipore (Billerica, MA). OCT medium was obtained from Tissue Tek Sakura (Torrance, CA).

### Experimental animals

Studies were approved by the North Carolina State University Institutional Animal Care and Use Committee. Breeding pairs of heterozygous mice (ClC-2^+/-^), a kind gift of Dr. James E. Melvin (University of Rochester, Rochester, NY), were used to generate ClC-2^-/-^ mice as described previously [39]. ClC-2^-/-^ and WT mice were identified as previously described [37,39] and 9-10-week-old mice were used unless indicated otherwise.

### Histology, immunohistochemistry, electron microscopy, and immunogold electron microscopy

Gastric tissues were collected in 10% neutral buffered formalin for histological evaluation. Tissues were sectioned (5 μm) and stained with hematoxylin and eosin or periodic acid schiff-alcian blue (PAS-AB), using standard methods. For morphometric analyses, the images were processed with Sigmascan Pro 5.0 (Systat, San Jose, CA).

Immunohistochemistry for H/K ATPase in murine gastric tissues was performed by standard methods. Heat activated antigen retrieval was performed in sodium citrate buffer (pH 7.4). Following inhibition of endogenous peroxidase and blocking in normal goat serum, the sections were incubated in 1:1000 mouse monoclonal anti-H/K ATPase β1 subunit antibody overnight at 4°C followed by 1:500 of the appropriate biotinylated secondary antibody for 1h at room temperature, then detected using horse radish peroxidase coupled avidin and peroxidase developing solutions.

For electron microscopy, stomach tissues were fixed in McDowell and Trump 4F:1G fixative and processed for transmission electron microscopy by standard techniques, as previously described [40]. In brief, after two rinses in 0.1 M sodium phosphate buffer (pH 7.2), samples were placed in 1% osmium tetroxide in the same buffer for 1 h at room temperature. Samples were rinsed twice in distilled water and dehydrated in an ethanolic series culminating in two changes of 100% acetone. Tissues were then placed in a mixture of Spurr resin and acetone for 30 min, followed by 2 h in 100% resin with two changes. Finally, samples were placed in fresh 100% resin in molds and polymerized at 70°C for 8 h to 3 days. Semi-thin sections (0.25–0.5 μm) were cut with glass knives and stained with 1% toluidine blue-*O* in 1% sodium borate. Ultrathin (70–90 nm) sections were cut with a diamond knife, stained with methanolic uranyl acetate followed by lead citrate, and examined with a transmission electron microscope (Phillips/FEICO model 208s, Hillsboro, OR). Transmission electron microscopic examination and imaging were carried out blind by an individual not directly associated with the study.

For immunogold microscopy, the ultrathin sections were incubated with 1:100 rabbit ClC-2 antibody as used by others [41] for 1h at room temperature followed by washings. The sections were then incubated with 1:50 rabbit IgG gold secondary antibody for 1 h followed by washings. The stained sections were then subjected for cutting and examination as indicated above for transmission electron microscopy.

### Gel electrophoresis and western blotting

Gastric tissues from WT and ClC-2^-/-^ mice were snap frozen and stored at −70°C. Tissue aliquots thawed at 4°C were added to chilled lysis buffer, containing protease inhibitors: 0.5 mM Pefabloc, 0.1 mM 4-nitrophenyl phosphate, 0.04 mM glycerophosphate, 0.1 mM Na_3_VO_4_, 40 μg/ml bestatin, 2 μg/ml aprotinin, 0.54 μg/ml leupeptin, and 0.7 μg/ml pepstatin A and homogenized on ice. After centrifugation at 2,000 x g for 10 min at 4°C the supernatant was collected and assayed for protein using a BCA Protein Assay Kit. Tissue lysates (amounts equalized by protein concentration) were mixed with equal volumes of 2 × SDS-PAGE sample buffers and boiled for 4 min. Lysate proteins were separated by SDS-PAGE on a 10% gel and transferred to a PVDF membrane. The membranes were blocked at room temperature for 2 h in Tris-buffered saline containing 0.05% Tween 20 (TBST) and 5% dry powdered milk, and then incubated overnight at 4°C with 1:1000 mouse monoclonal anti-H/K ATPase-β1 subunit, 1:200 rabbit anti-ClC-2 and 1:40,000 rabbit anti-β-actin antibodies. After washings in TBST, membranes were incubated with 1:5000 horseradish peroxidase conjugated secondary antibody for 1h, washed with TBST and developed for visualization of protein with luminol enhancer solution. Protein expression in western blots was semi-quantified using densitometric analyses (SigmaScan Pro, Systat, San Jose, CA) and was normalized to β-actin, the loading control.

### Immunofluorescence and confocal microscopy

Gastric tissues were embedded in OCT medium, frozen, sectioned at 5 μm, and stored at −80°C until use. The sections were thawed, fixed in cold acetone, and blocked with 10% normal goat serum for 60 min at room temperature. The sections were incubated overnight at 4°C with single or double combinations of primary antibodies diluted in 2% normal goat serum: 1:100 H/K ATPase β1 subunit and 1:100 ClC-2. After washes with PBS, the sections were incubated for 1 h at room temperature with 1:500 appropriate secondary antibodies conjugated with Alexa Fluor 488 (green) or Cy3 (orange) diluted in 5% normal goat serum. After washing with PBS, mounting medium containing DAPI (nuclear stain, blue) was added. The slides were examined with a Nikon Eclipse 2000E inverted microscope equipped with the Nikon C1 confocal laser scanning system. Cy3 was assigned the color red using the confocal microscope which is able to detect and reassign color to facilitate contrast.

### Measurement of gastric content pH

Fasted WT and ClC-2^-/-^ mice were injected subcutaneously with PBS or histamine (2 μg/g body wt) in PBS, euthanized 15 min and pH of the gastric contents was carried out as previously described [34,42]. The pHs were converted to [H^+^].

### Measurement of acid and pepsinogen secretion rates

Adult WT and ClC-2^-/-^ (10–20 wks, 20–40 g) were fasted for 2 h, anesthetized with halothane followed by 150 mg/kg thiobutabarbital IP and the trachea was cannulated. Fasting for 2 h prior to the experiment gave similar results to those obtained after overnight fasting. This was likely due to the 30 min perfusion to clear the stomach contents before the experiment was started and an additional 30 min perfusion during which 15 min collections were made prior to secretagogue addition. The gastric mucosa was perfused as previously described in detail [43] using a tube fed into the stomach via the esophagus and a tube fed back into the stomach from the duodenum and continuously perfused at a rate of 16 ml/h. When the gastric contents of the stomach were cleared (about 30 min), the gastric effluent was then collected every 15 min. At 30 min, subcutaneous infusion of 0.23 mg/h histamine and 0.03 mg/h diphenhydramine (H_1_ blocker) was started and the gastric perfusate was changed to contain 0.5 mg/ml carbachol. This combination of histamine, carbachol and gastric perfusion was used because this procedure resulted in the most reproducible maximal acid secretory response in mice [27]. Gastric effluent samples were analyzed for acid by back titration, for pepsinogen using the method of Anson and Mirsky [44] and pH was measured. Since maximum acid secretion rates were variable as previously reported [27], results were normalized to pepsinogen secretion. When pepsinogen was <18,000 units/45 min of stimulation, the tissue was excluded since it was likely damaged or compromised. This criterion was used with both WT and ClC-2^-/-^ mice.

### Statistical analysis

Data are reported as means ± SE. Statistical significance was calculated using the Students unpaired t test.

## Results

### Histological characterization of the gastric mucosa of WT and ClC-2^-/-^ mice

**Fig 1:**
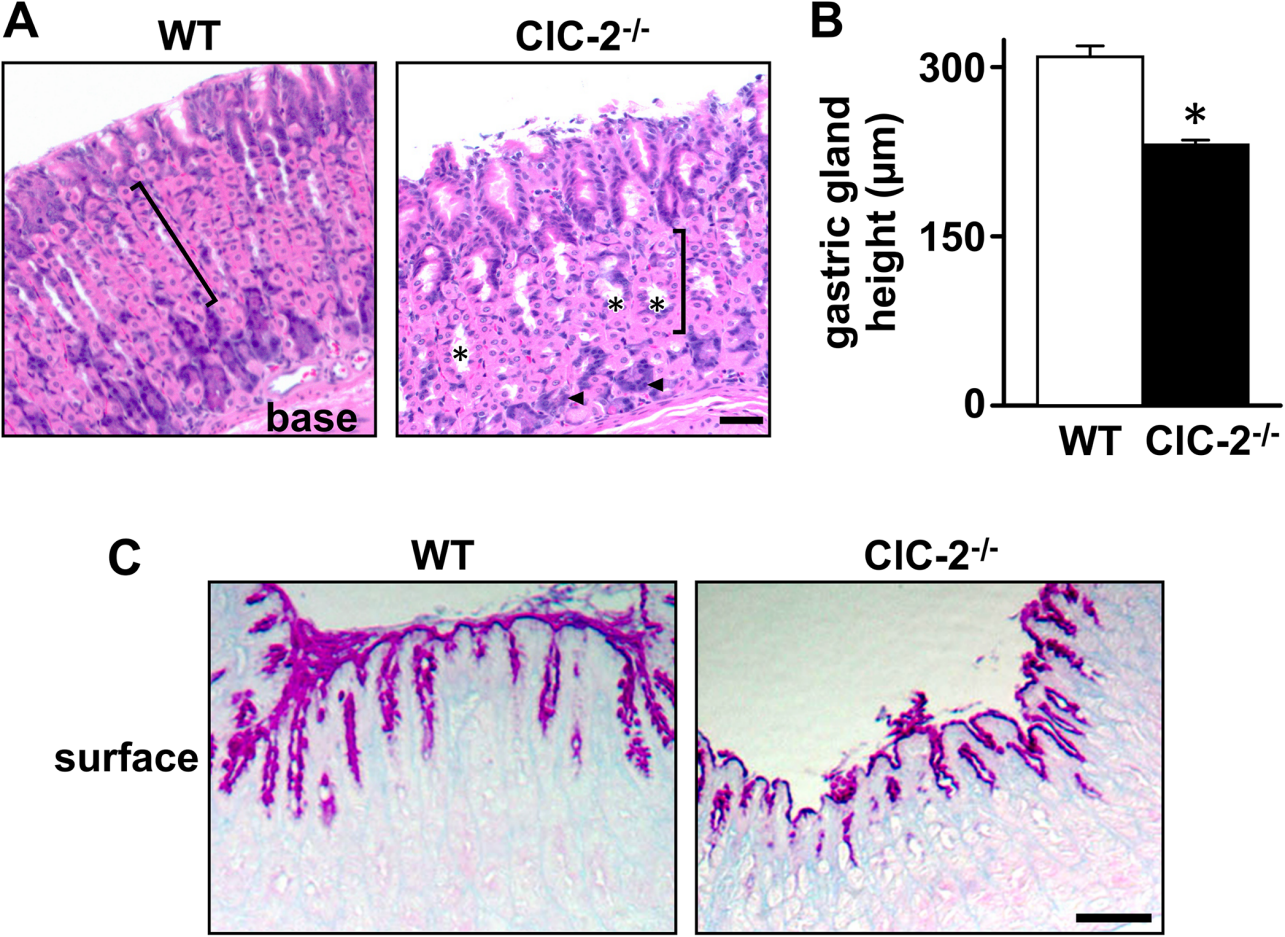
Histological characterization of the gastric mucosa of WT and ClC-2^-/-^ mice. **A.** Haematoxylin and eosin staining of the gastric mucosa of WT and ClC-2^-/-^ mice is shown. Flat pink cells with purple nuclei are parietal cells; dark purple cells are zymogen cells (arrowheads). Brackets indicate the gastric gland layer and * indicates places of glandular dilation. Bar = 50 μm. **B.** Height of the gastric gland region was measured in WT and ClC-2^-/-^ mice. Results are plotted as mean ± SE (n = 6). *P<0.001 versus WT. **C.** PAS-AB staining of mucus cells in WT and ClC-2^-/-^ mouse gastric mucosa. Neutral mucin positive surface mucus cells are dark pink and parietal cells show faint pink staining. Bar = 25 μm.

Histological sections of the gastric mucosa from WT and ClC-2^-/-^ mice were examined. In H & E stained sections of young mice (9-weeks-old), the gastric mucosa of ClC-2^-/-^ mice showed dilation of the gastric glands and disorganization of cell layers, including surface mucous secreting cells, parietal cells, and zymogen cells, as compared to the well orientated, organized cell pattern in WT mouse gastric mucosa (Fig 1A). There appeared to be reduced numbers of parietal cells in the glands (Fig 1A) and the height of the gastric gland region of the gastric mucosa was significantly reduced by 24.4% (*P*<0.001) in ClC-2^-/-^ mice as compared to WT mice (Fig 1B). Similar histological changes were also present in the stomachs of older ClC-2^-/-^ mice (11-month-old, data not shown). As shown in Fig 1C, PAS-AB-stained sections showed an extensive branched pattern of mucus positive cells in WT stomach, whereas staining was minimally branched and superficial in ClC-2^-/-^ stomach.

### H/K ATPase expression in the gastric mucosa of WT and ClC-2^-/-^ mice

**Fig 2:**
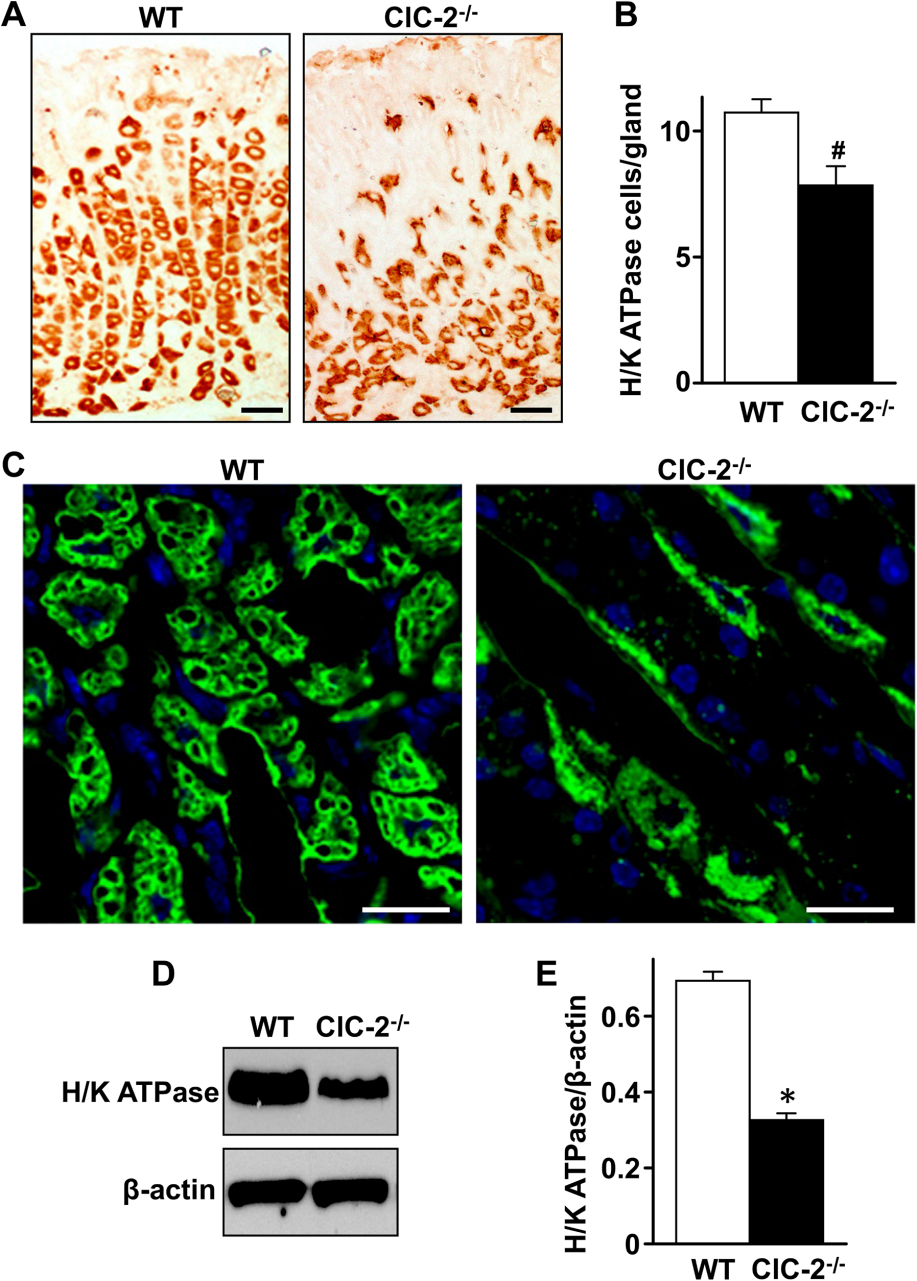
Immunolocalization and expression of H/K ATPase-β subunit in WT and ClC-2^-/-^ mouse gastric mucosa. Gastric mucosal sections from WT and ClC-2^-/-^ mice were stained for H/K ATPase β subunit by immunohistochemistry (A) and immunofluorescence (C). H/K ATPase β subunit positive cells were orange/brown in (A) and green in (C). Bar in (A) = 25 μm; bar in (B) = 10 μm, representative figures from n = 10–20 regions examined. (B) Quantitation of H/K ATPase-containing cells/gland of WT and ClC-2^-/-^ gastric mucosa, n = 6. #*P*<0.05 versus WT. (D) Western blot of H/K ATPase in WT and ClC-2^-/-^ mouse gastric mucosa, with β-actin as loading control. (E) Quantitation of H/K ATPase western blot by densitometry, normalized to β-actin, n = 3. *P<0.001 versus WT. Data in (B) and (E) are plotted as mean *±* SE.

To quantitate the change in the parietal cell population seen in Fig 1A, the expression of H/K ATPase protein was examined by immunohistochemistry, immunofluorescence and western blot analyses. In WT stomach examined by immunohistochemistry (Fig 2A), there was no staining of the surface epithelial layer, and abundant staining of the H/K ATPase within the parietal cells throughout the rest of the gastric mucosa. The parietal cells were well organized and most concentrated in the gastric gland region. However, in ClC-2^-/-^ mice, H/K ATPase staining was sparse and disorganized in the gastric gland region, more abundant but disorganized at the base of the mucosa and the cells appeared somewhat misshapen. Quantification of H/K ATPase positive cells showed that there was a significant 34.3% reduction (*P*<0.05) in the number of parietal cells per gastric gland in ClC-2^-/-^ mice (Fig 2B). Using immunofluorescent staining of H/K ATPase (Fig 2C), gastric gland parietal cells were abundant in WT and clearly reduced in ClC-2^-/-^ gastric mucosae. H/K ATPase protein expression was also analyzed by western blot with β-actin, the loading control, used for normalization (Fig 2D). H/K ATPase expression in ClC-2^-/-^ mouse gastric mucosa was significantly decreased (*P*<0.001) by 52.9% compared to WT (0.694 ± 0.023 in the WT and 0.327 ± 0.017 in ClC-2^-/-^ (n = 3)) shown in Fig 2E.

### Ultrastructure of parietal cells in WT and ClC-2^-/-^ gastric mucosa

**Fig 3:**
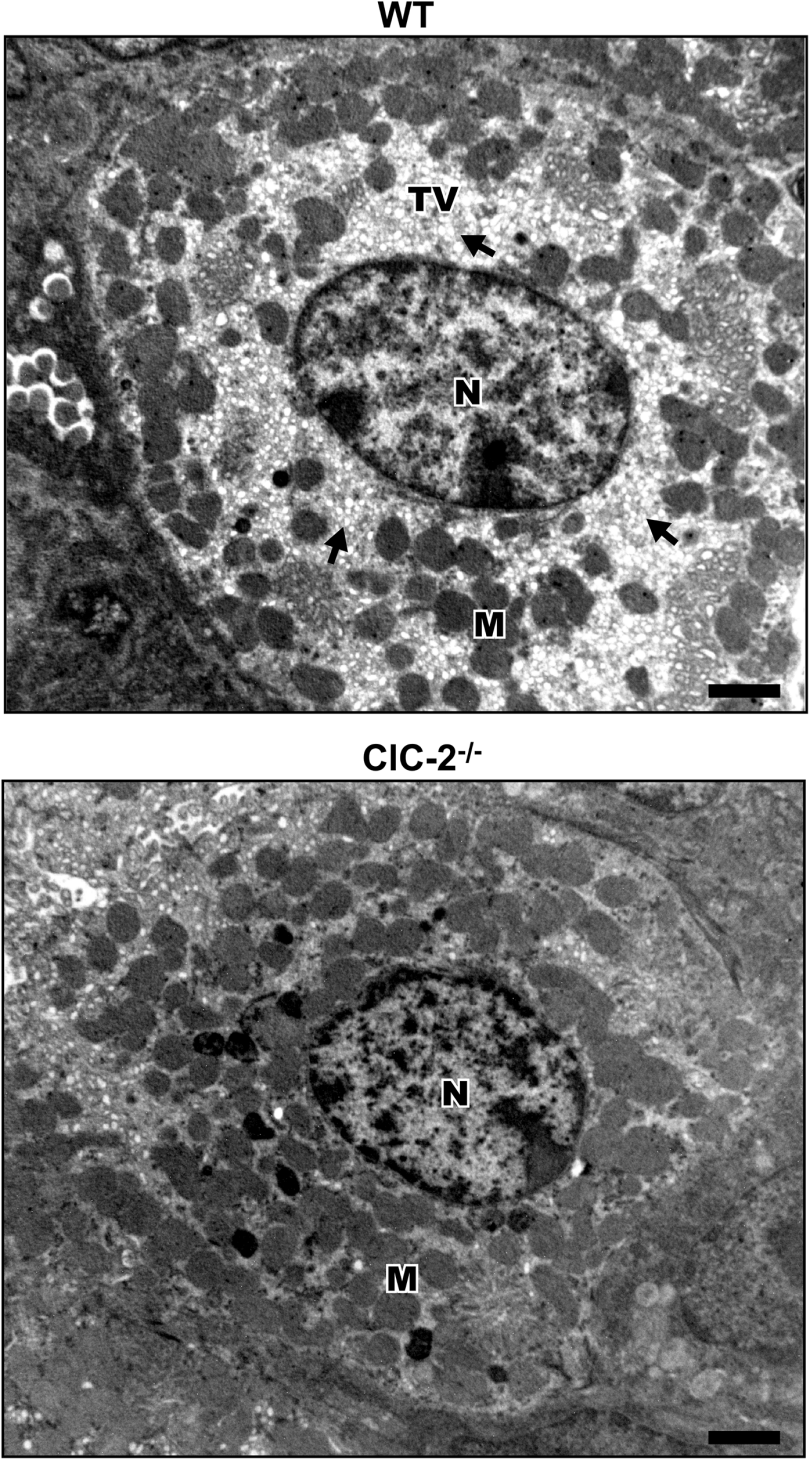
Ultrastructure of parietal cells in WT and ClC-2^-/-^ mouse gastric mucosa. Nucleus (N), mitochondria (M) and tubulovesicles (TV) are indicated. Electron dense bodies in the ClC-2^-/-^ panel are presumed to be fragments of mitochondria. Representation of n = 4 with at least 10 parietal cells examined from 3 different areas of each sample. Bar = 1 μm.

To examine whether the parietal cells present in ClC-2^-/-^ gastric mucosa albeit in reduced numbers, looked structurally normal, parietal cell ultrastructure in WT and ClC-2^-/-^ gastric mucosae was examined by electron microscopy. In similarly collected and processed WT and ClC-2^-/-^ mouse stomachs, parietal cells were identified based on the abundance of mitochondria and the presence of numerous small membrane bound tubulovesicles in the cytoplasm. As shown in Fig 3 both WT and ClC-2^-/-^ parietal cells had abundant mitochondria. However, compared to readily apparent and abundant tubulovesicles in the parietal cells of WT mouse stomach, parietal cells in ClC-2^-/-^ mouse stomach showed a reduced presence of tubulovesicles without any evidence of expanded canaliculi (Fig 3).

### Immunolocalization of ClC-2 with H/K ATPase in mouse parietal cells

**Fig 4:**
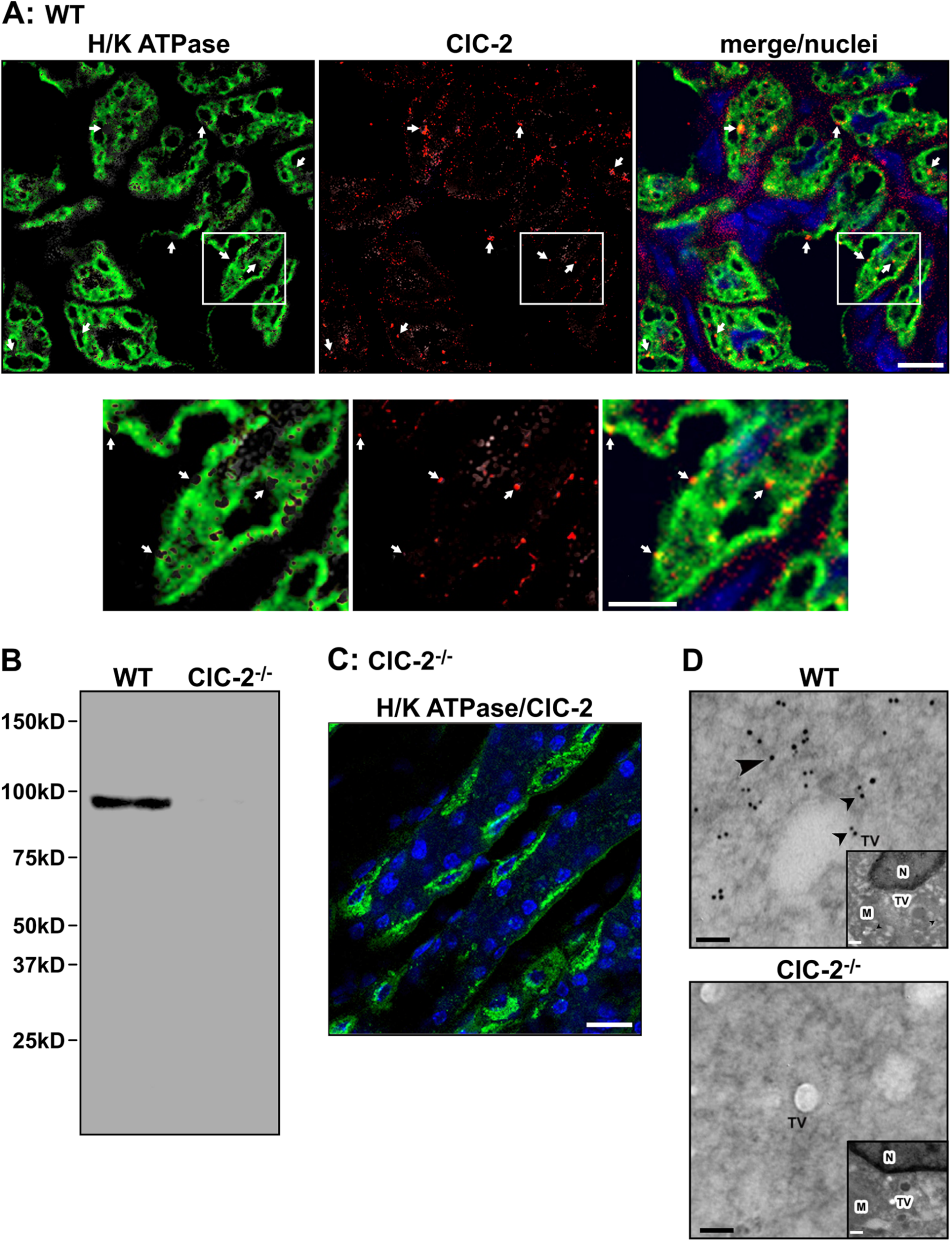
Immunolocalization and expression of ClC-2 in parietal cells of WT and ClC-2^-/-^ mouse gastric mucosa. (A) WT gastric mucosa was stained for both H/K ATPase (green) and ClC-2 (red). Nuclei are stained blue, bar = 25 μm. Lower panels show the area delineated by the white boxes magnified 2.5X, bar = 10 μm. (B) H/K ATPase (green) and ClC-2 (red) stained ClC-2^-/-^ gastric mucosa. Nuclei are stained blue, bar = 25 μm. (C) Western blot of ClC-2 in WT and ClC-2^-/-^ mouse gastric mucosa, with β-actin as loading control. Molecular weight markers are indicated. (D) Immunogold electron microscopy of ClC-2 in WT and ClC-2^-/-^ mouse gastric parietal cells. Gold labelling is seen as large black dots (black arrowheads). TV, tubulovesicles, bar = 100nm. Inset shows lower magnification of the parietal cell showing M, mitochondria, TV and N, nucleus for orientation, bar = 1 μm. Representative figures of n = 10–20 regions examined.

ClC-2 was localized in the gastric mucosa by immunofluorescent confocal microscopy. As shown in Fig 4A WT gastric mucosa showed diffuse H/K ATPase staining (green fluorescence) in the parietal cell cytoplasm and around the secretory canaliculi. ClC-2 (red fluorescence) was evident as numerous punctate and larger spots within the same area as the H/K ATPase staining. Fig 4B shows a western blot of WT and ClC-2^-/-^ gastric mucosa. A single ca. 98kDa protein band was evident in WT and absent from ClC-2^-/-^ mouse gastric mucosa. No additional bands are evident, indicating high specificity of the ClC-2 antibody. This is also shown in Fig 4C, which shows the control ClC-2^-/-^ gastric mucosa stained for ClC-2 and H/K ATPase. H/K ATPase (green fluorescence) in parietal cells was evident and the absence of red fluorescence indicated absence of ClC-2 and non-specific staining. The presence of ClC-2 in WT parietal cells was confirmed using immunogold electron microscopy (Fig 4D). For orientation, low magnification micrographs are shown as insets. In WT parietal cells ClC-2 appeared near/associated with tubulovesicles with ClC-2-linked gold particles appearing as black dots, whereas no gold particles were evident in ClC-2^-/-^ parietal cells. This was also evident in the low magnification micrographs.

### Effect of histamine on the pH of gastric contents and effect of histamine/diphenhydramine/carbachol on acid secretion in WT and ClC-2^-/-^ mouse gastric mucosa

**Fig 5:**
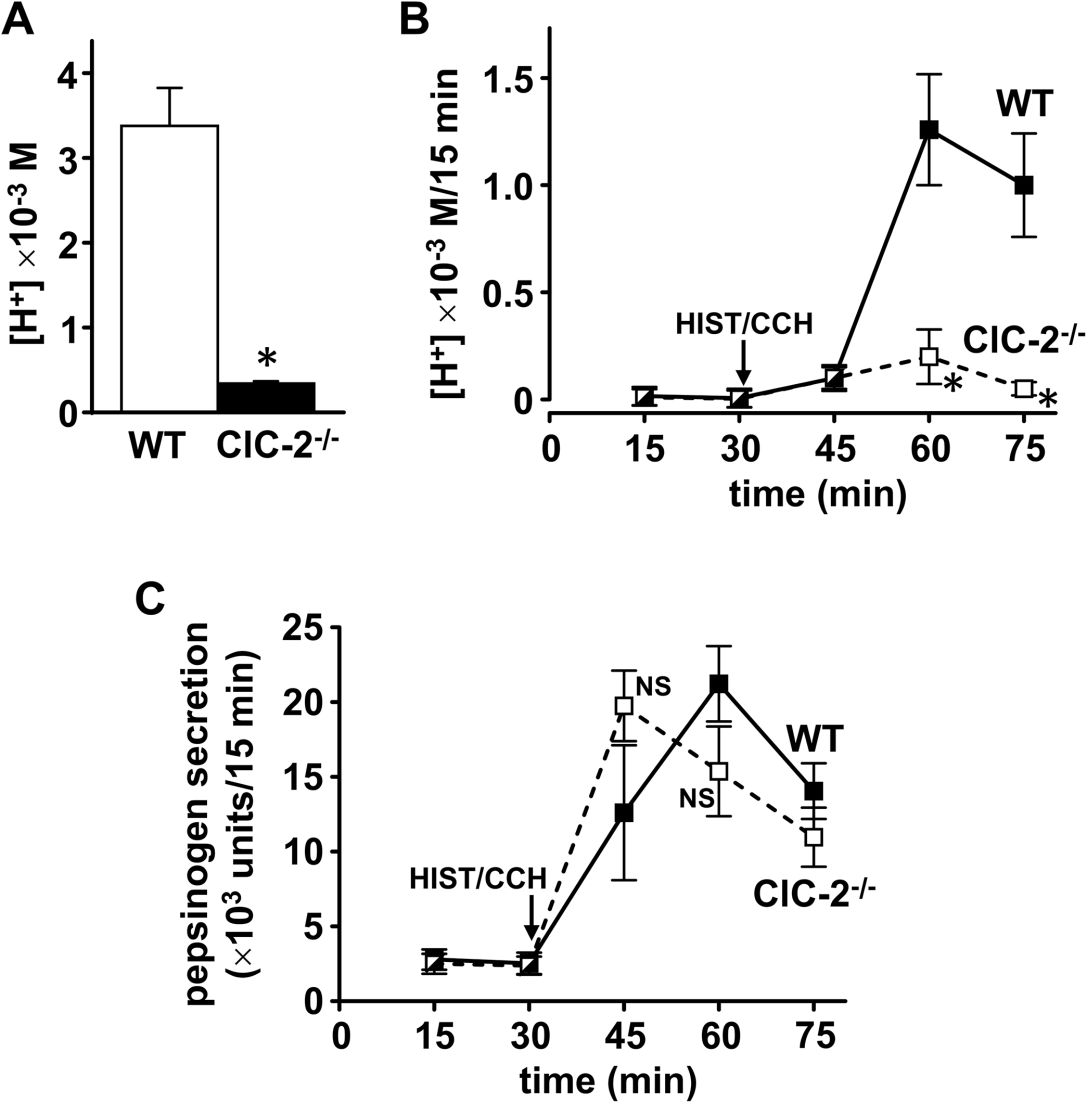
(A) Effect of histamine on the pH of gastric contents and (B) & (C) effect of histamine/carbachol on acid (B) and pepsinogen (C) secretion in WT and ClC-2^-/-^ mouse gastric mucosa. (A) The pH of gastric contents was measured in WT (white column) and ClC-2^-/-^ (black column) mice after 15 min of histamine stimulation. Data are plotted as mean ± SE (n = 3). *P<0.025 versus WT. For (B) & (C) mouse stomachs were perfused and at 30 min subcutaneous histamine/diphenhydramine (HIST, 0.23 mg/h/DPH, 0.03 mg/h) and intraluminal carbachol (CCH, 0.5 mg/ml) were started. 15 min samples of gastric effluent were collected and acid, pH and pepsinogen were measured. Data are plotted as mean ± SE. For (B) WT n = 11 & ClC-2^-/-^ n = 8, *P<0.005 versus WT. For (C) WT & ClC-2^-/-^ n = 7, NS, not significant versus WT.

In view of altered gastric mucosal organization/morphology, fewer parietal cells, reduced H/K ATPase expression and reduced tubulovesicles in ClC-2 ^-/-^ parietal cells, the pH of the gastric contents before and after histamine and acid secretion stimulated with histamine/carbachol were both measured in WT and ClC-2^-/-^ mice. The pH of the gastric contents measured before (basal) and after 15 min of histamine stimulation were 4.97 ± 0.07 and 2.30 ± 0.05 in WT and 6.43 ± 0.03 and 3.30 ± 0.03 in ClC-2^-/-^ mice (n = 3). Converting the pH values to [H^+^], the effect of histamine on [H^+^] is shown in Fig 5A. Histamine stimulated gastric content [H^+^] was 9.4-fold (89.3%) (*P*<0.025) decreased in ClC-2^-/-^ mice compared to WT mice (WT 5.1 ± 0.7 x 10^−3^ M (n = 3), ClC-2^-/-^ 0.55 ± 0.04 x 10^−3^ M (n = 3)). Basal gastric content [H^+^] was extremely low: 0.011 ± 0.002 x10^-3^ M in WT and 0.00037 ± 0.00003 x 10^−3^ M in ClC-2-/-, compared to histamine stimulated gastric content [H^+^]. These values were 0.22% and 0.07% of maximum [H^+^] respectively and were significantly different from each other at *P*<0.05.

Acid secretion rates in WT and ClC-2^-/-^ perfused gastric mucosae stimulated with histamine and carbachol were also measured. Acid secretory rates in the WT were similar to those reported by Schofield, Ito & Bolender [27] and maximal acid secretion occurred at 30 min after addition of secretagogues. For WT (n = 11), acid secretion in μEq/15 min was 0 min (basal), 0.95 ± 0.33; 15 min, 3.31 ± 0.99; and 30 min, 8.71 ± 1.39. For ClC-2^-/-^ (n = 8), acid secretion in μEq/15 min was 0 min (basal), 0.32 ± 0.08; 15 min, 1.77 ± 0.39; and 30 min, 1.6 ± 0.60. There was a 3.5-fold significant increase in acid secretion after 15 min of histamine/carbachol (*P*<0.025 for WT; P<0.005 for ClC-2^-/-^). Histamine/carbachol stimulated acid secretion in ClC-2^-/-^ gastric mucosa was greatly reduced compared to WT: decrease was 46.5% at 15 min, 63.3% at 30 min and 78.2% at 45 min. Basal acid secretion was very low: 0.22% and 1.5% of maximal acid secretion at 30 min in WT and ClC-2^-/-^, and they were significantly different (*P*<0.05).

Acid secretion expressed as [H^+^] calculated from pH measurements is shown in Fig 5B. The increase in acid secretion after 15 min of histamine/carbachol was significant (*P*<0.05) for WT gastric mucosa, but not significant for ClC-2^-/-^. Histamine/carbachol stimulated acid secretion was significantly reduced in ClC-2^-/-^ gastric mucosa compared to WT (*P*<0.005) with decreases of 84% and 95% at 30 and 45 min respectively. Basal [H^+^] was very low: 0.006 ± 0.003 (n = 11) in WT and 0.003 ± 0.002 (n = 8) in ClC-2^-/-^ x10^3^ M/15 min, 0.5% and 1.5% of maximum [H^+^] after 30 min of histamine/carbachol respectively and not significantly different from each other. Fig 5C shows pepsinogen secretion before and after histamine/carbachol treatment. There was no difference in pepsinogen secretion in WT compared to ClC-2^-/-^ gastric mucosa over a similar time course.

## Discussion

The aim of the present study was to investigate whether genetic ablation of ClC-2 has any effects on the gastric mucosa with a focus on parietal cell abundance, H/K ATPase expression, morphology and acid secretion using WT and ClC-2^-/-^ mouse gastric mucosa. It was suggested that ClC-2 is important for gastric parietal cell acid secretion [3,4,33]. However, others concluded that ClC-2 was not involved in gastric acid secretion [34] since the pH of both WT and ClC-2^-/-^ gastric stomach contents was similar with and without histamine. No other aspect of gastric parietal cell physiology was examined.

In the present studies ClC-2^-/-^ gastric mucosa had marked histological/morphological alterations that included dilation of the gastric glands, reduced height of the gastric gland region by 24%, and disorganization of surface mucus cell, parietal cell and zymogen cell layers. In addition numbers of parietal cells were significantly reduced by 34% and expression of H/K ATPase was also significantly reduced by 53%, likely due in part to the decrease in parietal cell number. ClC-2 was present in WT mouse gastric parietal cells in the same area of the parietal cell as the H/K ATPase confirming previous observations [33]. Using immunogold electron microscopy, ClC-2 appeared associated with parietal cell tubulovesicles. Examination of the ultrastructural features of ClC-2^-/-^ parietal cells compared to WT showed a marked reduction in the presence of tubulovesicles without evidence of expanded canaliculi. Tubulovesicles are important membrane structures that allow rapid and robust activation followed by cessation of acid secretion [26,27].

Reduction of parietal cell numbers, reduced H/K ATPase expression and reduced tubulovesicles in the ClC-2^-/-^ gastric mucosa each could separately and together lead to reduced acid secretion. In the present studies using similar methods as others [34], ClC-2 knockout resulted in significant 89% decreases (*P*<0.025) in [H^+^] of gastric contents compared to WT after 15 min of histamine stimulation from 5 to 0.5 x 10^−3^ M. In WT mice when the stomach was perfused, histamine/carbachol greatly increased acid secretion to levels similar to those previously reported [27]. In contrast, in ClC-2^-/-^ mice, histamine/carbachol resulted in greatly and significantly reduced acid secretion by 95% (*P*<0.005), while there was no effect on gastric pepsinogen secretion, which was similar in WT and ClC-2^-/-^ mice. Basal [H^+^] although present, was very small in both WT and ClC-2^-/-^ gastric contents and gastric perfusion experiments. Although in gastric contents experiments, basal [H^+^] was significantly different at *P*<0.05 in ClC-2^-/-^ compared to WT; in gastric perfusion studies there was no significant difference between WT and ClC-2^-/-^ basal [H^+^]. Time course of these two types of experiments cannot be closely compared due to variability in the perfusion studies and differences in the time frame for removal of stomach contents. However there is a significant increase over basal in histamine/carbachol stimulated acid secretion at 15 min in WT but not in ClC-2^-/-^ as shown in Fig 5B and when measured as μEq/15 min WT acid secretion (μEq/15 min) increased 3.5-fold over basal.

Mice with H/K ATPase α-subunit, H/K ATPase β-subunit, anion Cl^-^/HCO_3_^-^ exchanger Slc26a9, Na/H exchanger NHE2 or Huntingtin interacting protein 1 (Hip1r) genetically ablated [25,42,45–48] all showed some similar changes in the gastric parietal cell as found in the present studies, including reduced presence of tubulovesicles and effects on acid secretion. Only ablation of the H/K ATPase α or β subunits or NHE2 (in adult mice, but not in juvenile mice) resulted in total absence of acid secretion (achlorhydria, no basal secretion), where the gastric content pH was ~7. With ablation of Slc26a9 or Hip1r proteins, stimulated acid secretion was reduced, but not abolished. It is interesting to note that ablation of Slc26a9 [30] reduced ClC-2 expression. Thus, each of these proteins as well as ClC-2 based on data shown in the present study appears to play an important role in gastric acid secretion. It is not possible from ClC-2 knockout studies to determine whether ClC-2 plays a direct role in acid secretion (to provide the Cl^-^ equivalents for HCl secretion) because of the many changes that occur, including reduced parietal cell numbers, reduced H/K ATPase and reduced tubulovesicles, which by themselves would be expected to reduce acid secretion. In the present studies the continued presence of very low basal [H^+^] in ClC-2^-/-^ mouse stomachs may suggest that channels/transporters other than ClC-2 may be responsible for this.

In conclusion, ablation of ClC-2 resulted in gastric gland dilation, reduced height of the gastric gland region (24%), disorganized cell layers in the gastric mucosa, loss of parietal cells (34%), reduced parietal cell H/K ATPase (53%), reduced parietal cell tubulovesicles without expanded canaliculi and reduced stimulated gastric acid secretion whether measured by monitoring the pH of the gastric contents or by gastric perfusion.

## Abbreviations

WT: ClC-2 wild type
ClC-2^-/-^: ClC-2 knockout
